# Tumor Endothelium Marker-8 Based Decoys Exhibit Superiority over Capillary Morphogenesis Protein-2 Based Decoys as Anthrax Toxin Inhibitors

**DOI:** 10.1371/journal.pone.0020646

**Published:** 2011-06-02

**Authors:** Chenguang Cai, Jinjing Che, Long Xu, Qiang Guo, Yirong Kong, Ling Fu, Junjie Xu, Yuanguo Cheng, Wei Chen

**Affiliations:** 1 State Key Laboratory of Pathogen and Biosecurity, Beijing Institute of Microbiology and Epidemiology, Beijing, China; 2 Laboratory of protein engineering, Beijing Institute of Biotechnology, Beijing, China; National Institutes of Health, United States of America

## Abstract

Anthrax toxin is the major virulence factor produced by *Bacillus anthracis*. The toxin consists of three protein subunits: protective antigen (PA), lethal factor, and edema factor. Inhibition of PA binding to its receptors, tumor endothelium marker-8 (TEM8) and capillary morphogenesis protein-2 (CMG2) can effectively block anthrax intoxication, which is particularly valuable when the toxin has already been overproduced at the late stage of anthrax infection, thus rendering antibiotics ineffectual. Receptor-like agonists, such as the mammalian cell-expressed von Willebrand factor type A (vWA) domain of CMG2 (sCMG2), have demonstrated potency against the anthrax toxin. However, the soluble vWA domain of TEM8 (sTEM8) was ruled out as an anthrax toxin inhibitor candidate due to its inferior affinity to PA. In the present study, we report that L56A, a PA-binding-affinity-elevated mutant of sTEM8, could inhibit anthrax intoxication as effectively as sCMG2 in Fisher 344 rats. Additionally, pharmacokinetics showed that L56A and sTEM8 exhibit advantages over sCMG2 with better lung-targeting and longer plasma retention time, which may contribute to their enhanced protective ability *in vivo*. Our results suggest that receptor decoys based on TEM8 are promising anthrax toxin inhibitors and, together with the pharmacokinetic studies in this report, may contribute to the development of novel anthrax drugs.

## Introduction

Anthrax toxin is the major virulence factor produced by *Bacillus anthracis* and consists of three protein subunits: protective antigen (PA), lethal factor (LF), and edema factor (EF). This toxin enters the cell cytoplasm and exerts toxic effects in a PA-mediated manner. More specifically, PA binds to cell surface receptors, forming a pre-pore complex after activation by furin at the cell membrane, followed by the binding of up to three molecules of LF and/or EF to the complex [1]. The entire complex is then internalized by receptor-mediated endocytosis [2]. Acidification in the endosome promotes transformation of the pre-pore complex into the pore complex and translocation of the catalytic LF and/or EF molecules into the cell cytosol.

The anthrax toxin receptors, tumor endothelium marker-8 (TEM8) [3] and capillary morphogenesis protein-2 (CMG2) [4], are type one transmembrane proteins that contain an extracellular von Willebrand factor type A (vWA) domain , which has been well established as the domain that directly interacts with PA [3], [4]. Other parts of the extracellular and transmembrane regions are necessary for anthrax intoxication, but the cytoplasmic region does not seem to be required [5]. However, cytoplasmic tails could regulate the vWA domain's affinity for PA binding and are important for efficient toxin uptake [2], [6], [7]. The highly conserved MIDAS motif in the vWA domain has been shown to be the key site for metal ion-dependent interactions with PA D683 [8]. Although their vWA domains share 60% identical residues, the two receptors significantly differ in their binding to PA: the 153–154 site, residing in the β4-α4 loop of CMG2, presents an additional interaction with PA domain 2 that does not occur with TEM8 [9].

Inhibition of PA binding to cell receptors has proven to be an effective therapy for anthrax intoxication. In addition to antibodies [10] and polyvalent molecules [11] targeted to the binding sites of PA or its receptors, soluble fragments of receptors, such as the mammalian cell-expressed vWA domain of CMG2 (sCMG2), have also been reported to inhibit PA-receptor binding [12]. Moreover, antibody Fc fragments have been fused to sCMG2, which efficiently improved their plasma residence time and preserved their affinity [13], [14]. Furthermore, the ability of sCMG2 to block antibody-resistant forms of anthrax toxin and relevant bacterial strains has been validated [13]. In addition, a new plant expression system has been built for producing Fc-fused CMG2 [14], [15]. However, because of its lower affinity, the vWA domain of TEM8 (sTEM8) was ruled out from the first antitoxin design [12]. Thus far, TEM8 in Fc fusion form has only been applied as an antitumor decoy [16].

In our previous work, we found that the replacement of the L56 residue in sTEM8 with the homologous alanine residue found in sCMG2 (referenced as L56A) could improve the antitoxin efficacy of sTEM8 in a cell-based anthrax toxin neutralization assay [17]. In the current study, we confirm the elevated affinity of L56A to PA and demonstrate its potency as a toxin inhibitor in rats. Pharmacokinetic studies were performed to compare the behaviors of sTEM8, L56A, and sCMG2 *in vivo*. The results demonstrate the advantages of sTEM8-based constructs over sCMG2 as anthrax toxin inhibitors.

## Results

### sTEM8 and its mutant L56A exhibit lower PA binding affinities than sCMG2

In our previous work [17], we demonstrated that the sTEM8 mutant L56A conferred increased protection to J774A.1 cells against anthrax lethal toxin (LeTx) challenge compared to wild-type sTEM8. To further evaluate the potency of L56A as an anthrax toxin inhibitor compared with sTEM8 and sCMG2 we prepared a new batch of proteins, repeated the cell protection assay and used an improved Schild plot analysis to compare the affinity of these constructs, as detailed in the experimental section.

**Figure 1:**
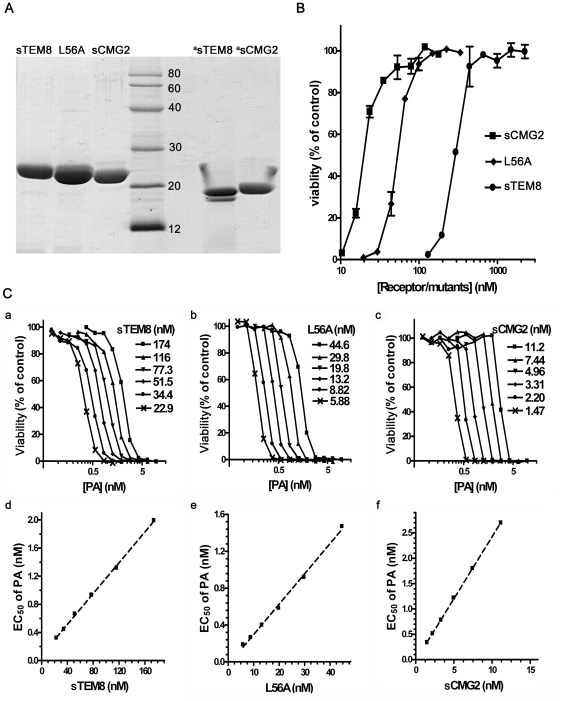
Receptor variants can protect J774A.1 cells from LeTx intoxication. *A*. vWA domains of anthrax toxin receptors/variants were fused with N-terminal His tags, purified by Ni affinity chromatography and anion exchange chromatography and analyzed with SDS-PAGE. *B*. The results for cell protection assays. Viability was assessed as described in the methods section. Each assay was performed at least three times, with duplicates within each assay. Data points represent the mean ± SEM in duplicate for one representative experiment. *C*. The results for the Schild plot assays; a, b, and c. PA dose-dependent survival curves for cells at a fixed receptor/mutant concentration; d, e, and f. These graphs were obtained by plotting the EC_50_ values corresponding to different receptor concentrations, fixed by linear regression using GraphPad Prism software. Each assay was performed at least three times. The data shown are for one representative experiment. ^a^ Samples were not mixed with 2-mercaptoethanol.

All the constructs were soluble in the cytoplasm of *Escherichia coli*. Without moving the His tag, there would be additional residues (MSHHHHHHSM) at the N-termini of the recombinant proteins. For all the purified proteins, only a single band was shown at a lower molecular weight than expected on non-reducing polyacrylamide gels (Fig. 1A), suggesting that the redundancy did not interfere with protein folding and the correct disulfide bond was formed, as the crystal structures showed [17].

The protection assay yielded results consistent with our previous study [17] with IC_50_ values of 274.6±8.7 nM, 69.5±5.8 nM, and 20.8±1.5 nM (Fig. 1B, Table 1) for sTEM8, L56A and sCMG2 respectively. sTEM8 and its more protective mutant L56A still performed less efficiently than sCMG2. Accordingly, the Schild plot analysis (Fig. 1C, Table 1) demonstrated that the sTEM8 mutant L56A elevated the affinity, with a Kd value 3.4 times lower than that of sTEM8, consistent with our previous results obtained by the BIAcore assays (29.8 nM for sTEM8 versus 4.44 nM for L56A) [17]. However, the affinity of L56A was still lower than that of sCMG2, with Kd values 11.1 times higher, confirming the results of the protection assay.

**Table 1:** Data results of the cell protection assay.

Receptor or mutant	IC_50_ (nM), Mean ± SEM	1/slope^a^
sTEM8	274.6±8.7	104.6±12.56
L56A	69.5±5.8	31.74±1.310
sCMG2	20.8±1.5	3.774±0.2914

a Reciprocal of the slope equals 
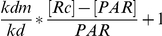
, used for comparison of the Kd values for receptors and mutants, as detailed in Supporting Information S1. Data represent the mean ± SEM values for three independent experiments.

### L56A exhibits similar or slightly better protection than sCMG2 *in vivo*

**Figure 2:**
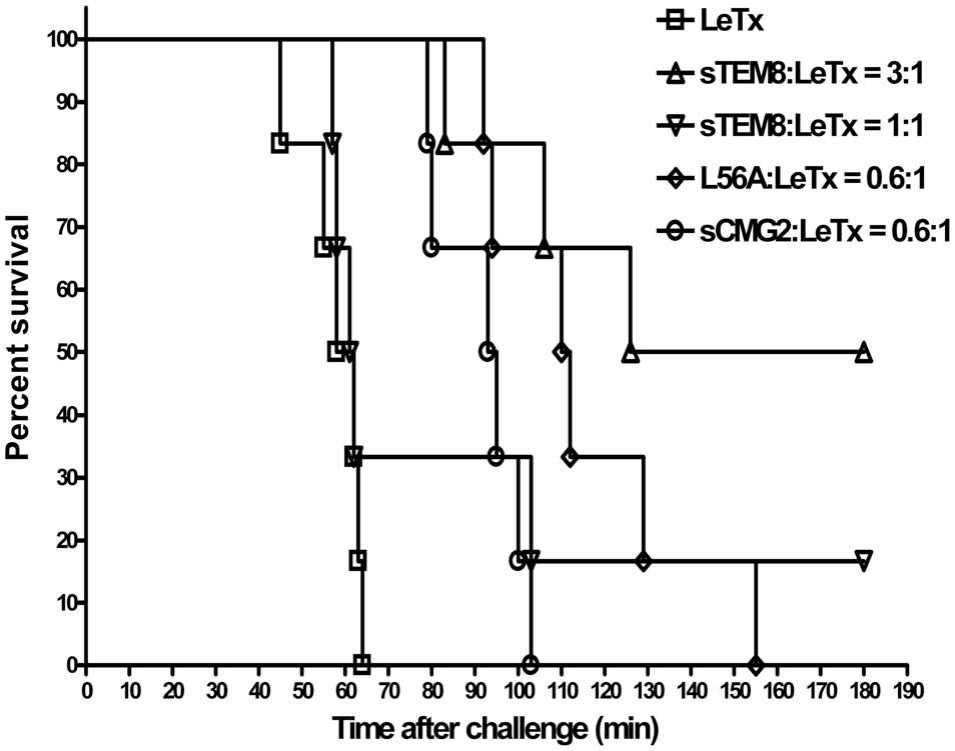
Receptor variants protect Rats from LeTx intoxication. Survival curves of rats protected by receptor decoys at different doses after anthrax lethal toxin attack. Ratios indicate the receptor/decoy∶ PA ratio when the former was mixed with lethal toxin (LeTx). Rats were monitored for 3 h after i.v. administration and then overnight. For convenient comparison, groups with full protection are not shown and are listed in Table 2.

Based on the ability of these receptor decoys to inhibit PA intoxication *in vitro*, we next tested the efficacy of sTEM8 and L56A compared to sCMG2 *in vivo*. Cell-produced sCMG2 has been reported to fully protect rats at a molar receptor∶ PA ratio of between 1∶1 and 2∶1, whereas cell-produced sTEM8 could not do so even at 15∶1 [12]. In our studies, the bacterially-produced sCMG2 exhibited similar efficacy (Table 2), conferring full protection at receptor∶ PA ratios of 3∶1 and 1∶1, and significantly increasing survival time at a ratio of 0.6∶1 compared with the LeTx-only group. Meanwhile, the bacterially-produced sTEM8 exhibited toxin inhibition superior to that of the reported cell-produced sTEM8, conferring full protection at receptor∶ PA ratios of 10∶1 and 5∶1, and protecting three of the six rats at a ratio of 3∶1 (Table 2). This difference was consistent with the BIAcore results (the prokaryote-expressed form has Kd values about 4 times lower than the eukaryote-expressed form: 29.8 nM versus 130 nM [17], [18], without considering the system errors). The difference between the two forms of sTEM8 may be ascribed to the different expression systems applied (with or without glycosylation, for example). As expected, the L56A mutant of sTEM8 performed better than sTEM8, conferring full protection at receptor∶ PA ratios of 3∶1 and 1∶1. Unexpectedly, however, L56A performed similarly to or slightly better than sCMG2 (with prolonged survival times of 115.3±9.660 min versus 91.67±4.112 min, respectively, at receptor∶ PA ratios of 0.6∶1. P = 0.0350, using the logrank test, GraphPad Prism software, San Diego California USA) (Fig. 2, Table 2).

**Table 2:** *In vivo* protection against intoxication provided by different receptor decoys.

Experiment, treatment (molar ratio^a^)	No. of survivors/total	Time to death (min)	P^b^
LeTx only^c^	0/6	58/62/63/64/45/55	
STEM8/LeTx:			
10∶1	3/3	NA	
5∶1	6/6	NA	
3∶1	3/6	126/106/83	0.0005
1∶1	1/6	62/103/57/61/58	0.3230
L56A/LeTx:			
3∶1	3/3	NA	
1∶1	6/6	NA	
0.6∶1	0/6	155/112/129/94/92/110	0.0005
SCMG2/LeTx:			
3∶1	3/3	NA	
1∶1	6/6	NA	
0.6∶1	0/6	100/95/103/80/79/93	0.0005

NA, not applicable.

a receptor decoy∶PA ratio when the latter is mixed with lethal toxin (LeTx).

b For comparison with the LeTx-only control group, by logrank test.

c LeTx-only control group (50 µg of PA and 25 µg of LF per rat).

### sTEM8 and L56A bind to plasma proteins with slower degradation rates than sCMG2 *in vivo*

**Figure 3:**
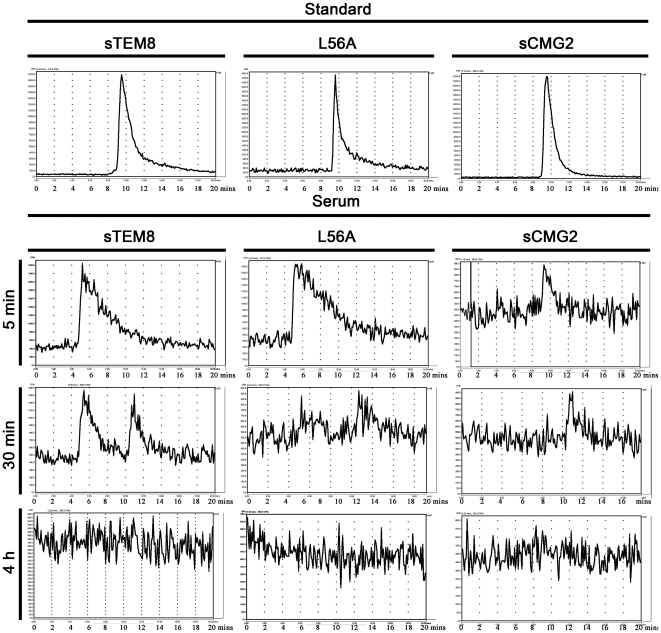
Plasma protein binding of the receptor decoys *in vivo*. Rats were i.v. injected with ^125^I-L56A, ^125^I-sTEM8, ^125^I-sCMG2 at a dose of 67.5 g/kg via the tail vein (at volume 250 µl). The serum samples collected at 5 min, 30 min, and 4 h after dosing were analyzed by size exclusion HPLC (TSK G3000 SWXL gel column)-flow scintillation analyzer (Radiomatic Model 600TRSeries, PerkinElmer, USA). For comparison, proteins labeled with ^125^I were loaded directly on columns as standards. When compared with the peak of the standard, a faster peak implied plasma protein binding, whereas a delayed peak may represent degraded protein.

sTEM8 and L56A had unexpectedly high efficacies *in vivo*. This finding suggests that they may have pharmacokinetics that differs from sCMG2. To address this hypothesis, we radiolabeled sTEM8, L56A, and sCMG2 with Na^125^I. Gel filtration monitored by radiation was used to test the chromatographic behaviors of the ^125^I-labeled proteins and the corresponding serum samples from rats after i.v. injection. The chromatography graphs are shown in Fig. 3. The retention time of the scintillation peaks could be used to estimate the size of the proteins (or their metabolites) with labeled radioactivity. Generally, shorter retention times imply increased molecular size caused by plasma protein binding, while prolonged elution times imply degradation.

There were no significant differences between the behaviors of the standard samples (^125^I-labeled proteins loaded directly). The three proteins all had single peaks with retention times slightly less than 10 min. After being injected i.v. into rats, sTEM8 and L56A exhibited comparable pharmacokinetics. The serum samples at 5 min showed only one peak, with a retention time of about 5 min. The clearly reduced retention times of the radiation peaks indicates that sTEM8 and L56A could bind to plasma proteins to form complexes with larger molecular weights. In contrast, a serum sample at 5 min showed a single peak for sCMG2 that was similar to that of its standard form, indicating a lack of plasma protein binding.

For the serum samples at 30 min, sTEM8 and L56A both showed two peaks: one with a retention time of about 5 min, similar to that of the serum samples at 5 min, likely representing the plasma protein binding complex, and another with a retention time of slightly more than 10 min, probably representing degraded metabolites. For sCMG2, only a single metabolite-peak with a prolonged retention time was observed. Comparing this with the chromatographic behaviors of the samples at 5 min suggests that sCMG2 degraded much faster than sTEM8 and L56A (Fig. 3).

### Pharmacokinetics of sTEM8, L56A, and sCMG2 indicate that all three proteins exhibit fast rates of elimination and extremely fast rates of distribution

**Figure 4:**
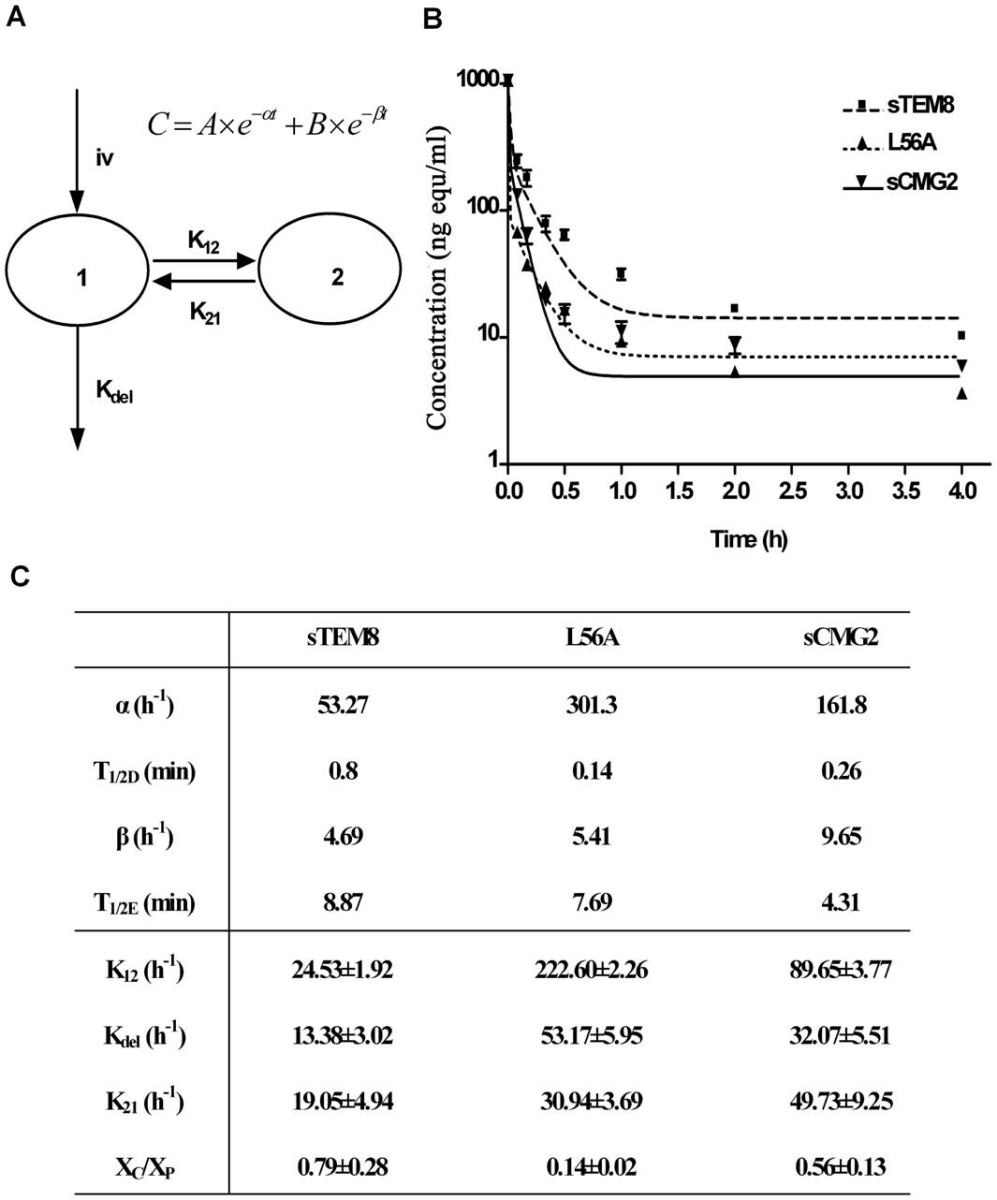
Pharmacokinetics of the variants. *A*. Schematic diagram of the two-compartment model, which is generally used for protein drugs administered i.v. by pulse injection. The two compartments are divided based on the different pharmacokinetic rates with the central compartment (denoted as compartment 1) representing the vascular cavity and the peri-compartment (denoted as compartment 2) representing relatively compact tissues. The elimination was limited in compartment 1 for simplification. The model can then be symbolized as 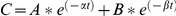. *B*. Plasma concentration-time plots of receptor decoys after i.v. injection into rats (n = 6) at a dose of 67.5 µg/kg. The point at time 0 was plotted based on a deduced initial concentration of 105 ng/ml, which equaled the injection amount (13.5 µg for 200 g on average) divided by the theoretical circulating blood volumes (12.8 ml for 200 g on average) [32]. Analysis applied the two-compartment model. Profiles are fitted by a two-phase exponential decay equation (GraphPad Prism software, San Diego California USA) with constraints of α>1, β>1, and values shared. *C*. Related pharmacokinetic parameters. Values for α, β were calculated by fitting plots in Fig. 4B with a two-phase exponential decay equation 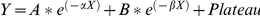 (GraphPad Prism software, San Diego California USA), with constraints of α>1, β>1, and values shared. Values for half time were calculated as 0.69/α and 0.69/β. The first degradation rate constants were calculated by 
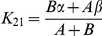
, 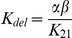 and 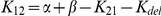. *K*_21_, and *K*_12_ represents the crossing rate between compartments, and *K*_del_ represents the excretion rate out of the system, as symbolized in figure 4A. X_C_/X_P_ represents the ratio of drug amount distributed in the central compartment to that of the peri-compartment at equilibrium, which equaled K_21_/K_12_.

The serum concentrations of ^125^I-sTEM8, ^125^I-L56A, and ^125^I-sCMG2 after i.v. injection at a 62.5 µg/kg dose were monitored over time and are plotted in Fig. 4B. The serum concentration profiles were fitted to a two-compartment model (Fig. 4A), and the related fitted curves are depicted in Fig. 4B with the corresponding pharmacokinetic parameters generated listed in Fig. 4C.

All three proteins exhibited fast elimination rates and much faster distribution rates, with an elimination half-life of less than 10 min and a distribution half-time of less than 1 min. For distribution, all three proteins were assigned higher values for K_12_ than K_21_, indicating greater distribution in the second compartment. The value of K_12_ for L56A was about 10-fold higher than that for sTEM8 (222.60 h^−1^ versus 24.53 h^−1^, Fig. 4C), and value of K_21_ was only about 2 times higher (30.94 h^−1^ versus 19.05 h^−1^, Fig. 4C), resulting in a dominantly higher distribution for the peri-compartment, as shown by values for X_c_/X_p_ (0.14 versus 0.79, Fig. 4C). Compared with sTEM8, sCMG2 exhibited about a 3.5-fold higher K_12_ value (89.65 h^−1^ versus 24.53 h^−1^, Fig. 4C) but about a 2.5-fold higher K_21_ (49.73 h^−1^ versus 19.05 h^−1^, Fig. 4C), resulting in a slightly higher distribution ratio (0.56 versus 0.79, Fig. 4C). The higher ratio for X_c_/X_p_ implies that L56A and sCMG2 are more likely than sTEM8 to remain in peri-compartments (such as target organs) but not central compartments (i.e., the circulation for body liquid), which may imply that their closer association with target organs contributes to their better performance.

For elimination, L56A exhibited the largest K_del_ value, which was even larger than that of sCMG2. However, when associated with the larger distribution rate K_12_, its elimination half-life was averaged to approach that of sTEM8. sCMG2 exhibited an elimination rate about 2.5-fold higher than sTEM8 (32.07 h^−1^ versus 13.38 h^−1^, Fig. 4C). When combined with its distribution rates, sCMG2 exhibited an elimination half-life of 4.31 min, which was about half that of sTEM8 (4.31 min versus 8.87 min, Fig. 4C).

### Tissue distributions show that sTEM8 and L56A target to the lungs, whereas sCMG2 targets to the kidney

**Figure 5:**
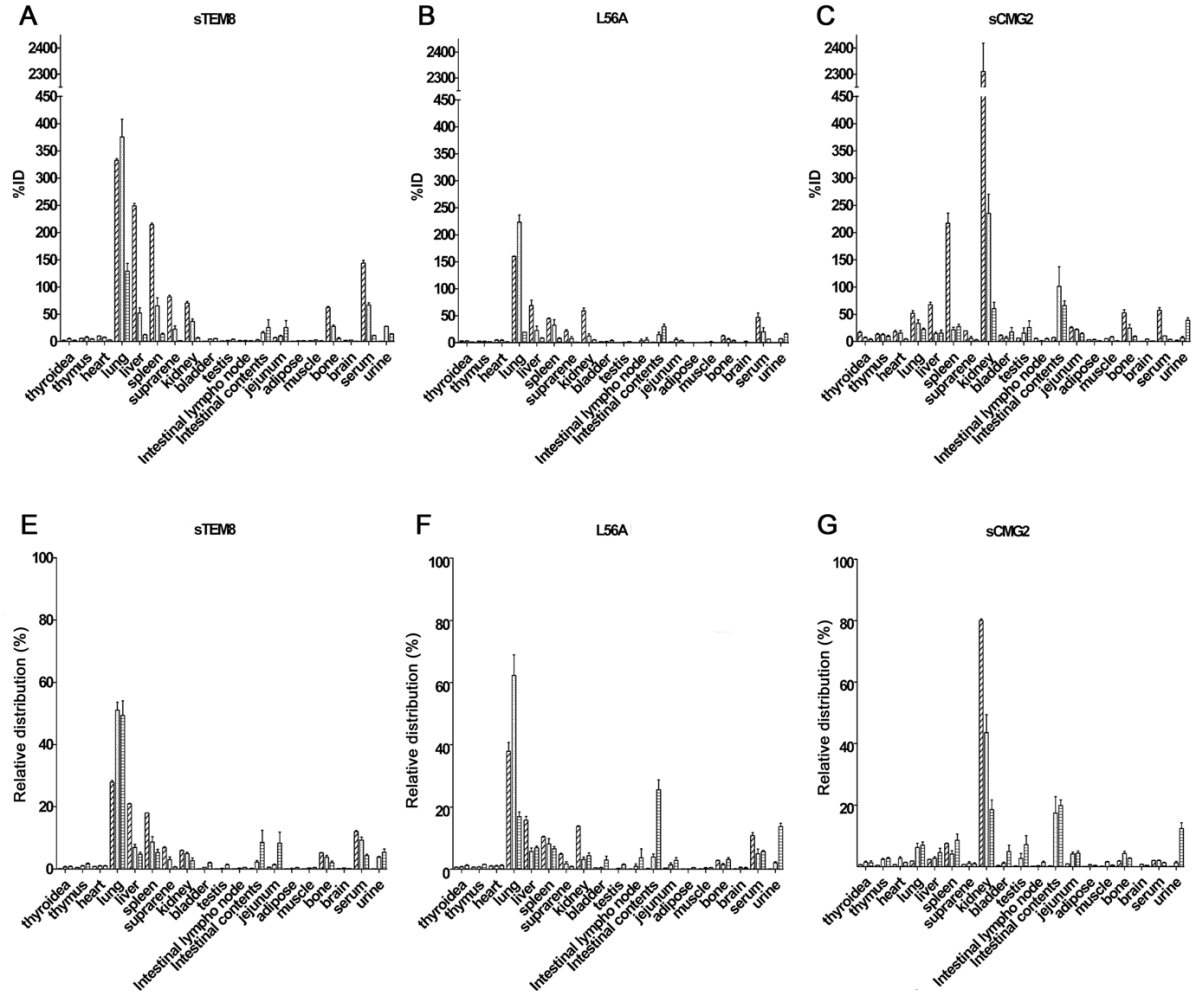
Tissue distribution of the variants. TCA-precipitable radioactivity (mean ±SEM) in various tissues determined at 5 min, 30 min, and 4 h (n = 6) after i.v. administration to rats. *A, B, and C*. %ID represents the equivalent drug concentrations calculated according to the radioactivity measured as a percentage of the deduced injection dose (67.5 ug/kg). *D, E, and F*. Relative distribution represents the drug concentration in a certain tissue as a percentage of the total drug concentration in all the tissues.

To compare the distribution of sTEM8, L56A, and sCMG2 in different tissues, TCA (Trichloroacetic Acid) sediments were investigated 5 min, 30 min, and 4 h after i.v. administration of a 67.5 µg/kg dose (Figs. 5).

The results indicated that all three proteins had wide distributions in tissues throughout the entire body within the time course examined. Most of the tissues already had significantly enriched TCA-precipitable radiations by 5 min after administration, especially the lung, liver, kidney, and spleen. This showed maintenance of higher protein concentrations compared with the serum, indicating a fast distribution rate for all three of the proteins (Fig. 5).

sTEM8 and L56A presented similar organ-targeting for the lung, which exhibited the highest observed concentrations: 332%, 375%, and 129% of the injected dose (ID) for sTEM8 at 5 min, 30 min, and 4 h, respectively (Fig. 5A), and 159%, 223%, and 19% for L56A (data sequenced) (Fig. 5B). By comparison, the concentrations in serum were 143%, 67%, and 11% for sTEM8 and 47%, 20%, 6% for L56A (Figs. 5A & 5B). When compared with other organs, the lung still received the largest fraction (presented as relative distribution in Figs. 5D & 5E); the concentration of sTEM8 in the lung accounted for 28%, 51%, and 49% of the sum of the concentrations of all the sampled tissues at 5 min, 30 min, and 4 h, respectively (Fig. 5D), and 37%, 62%, and 17% for L56A (Fig. 5E). By comparison, the relative distributions in the serum were 12%, 9%, and 4% for sTEM8 and 11%, 5%, and 6% for L56A (Figs. 5D & 5E). At all the sampled time points, the lung exhibited much higher concentrations than the serum for both sTEM8 and L56A. For sCMG2, the concentration in the lung was comparable to that in the serum (Figs. 5C & 5F) at 5 min, and much less than was found in the lung for sTEM8 and L56A, in terms of both the absolute amount (%ID) and the relative distribution at 5 and 30 min.

By contrast, sCMG2 exhibited a completely different distribution, targeting the kidney rather the lung: %ID values for the kidney were 2310%, 235%, and 60% of the injected dose (Fig. 5C), with relative distributions of 80%, 44%, and 19% at 5 min, 30 min, and 4 h, respectively. By comparison, %ID values for serum were 57%, 10%, and 4%, with relative distributions of 2%, 2%, and 1.2% (Fig. 5C). sTEM8 and L56A did not show any significant pooling in the kidney compared with the serum (Figs. 5A, 5B, 5D, & 5E), and the concentrations and relative distributions were much lower than for sCMG2 at all time points.

## Discussion

The comparable *in vivo* performance of L56A compared with sCMG2 is unexpected, considering its clearly lower potency observed in the *in vitro* assays (directly shown as IC_50_, 69.5±5.8 nM versus 20.8±1.5 nM), which was comparatively consistent with the apparent affinity detected (displayed as 1/slope, 31.74 versus 3.78, Table 1). Moreover, considering that the results of groups sTEM8/LeTX 3∶1 and L56A/LeTx 0.6∶1 did not show significant differences (p = 0.1514, logrank test, Fig. 2, Table 2), the relative *in vivo* performance of L56A versus sTEM8 was comparable to that *in vitro* (274.6 nM versus 69.5 nM, Table 1). The discrepancy between the *in vitro* and *in vivo* efficacy of the sTEM8-based decoys (sTEM8 and its mutant form L56A) and sCMG2 imply that inconsistencies occur after i.v. administration.

The size exclusion HPLC-flow scintillation analysis showed that sTEM8 and L56A exhibited an ability to bind plasma proteins, whereas sCMG2 did not. The analysis also indicated that sCMG2 may disrupt faster in plasma. The greater than 90% plasma protein binding for sTEM8 and L56A may be ascribed to their negative charge, which is predicted to be about −6.10 at pH 7.0 and is supported by the chromatography strategy used, as implied by the research on oligonucleotide pharmacokinetics [19]. By comparison, sCMG2 carries a positive charge of about 1.37 and did not bind to anion-exchange columns at near-neutral pH. However, measurements of the dependence of plasma binding on pH and ion strength are still needed to test this nonspecific binding hypothesis, although specific receptor-ligand interactions in the plasma seem unlikely [16], [20].

Tissue distribution studies showed that sTEM8 and L56A mainly target to the lung, whereas sCMG2 targets to the kidney but not the lung. sTEM8 and L56A contain a lung-targeting GFE motif, whereas sCMG2 contains a kidney-targeting DRG motif [21], [22], which may partially explain their differential targeting. However, multiple factors may contribute to organ specific distribution. For constructs with molecular weights of about 21 Kd, glomerular filtration in the kidney may be the main pathway of elimination. Plasma-binding may help sTEM8 and L56A escape this process to some extent. Certain receptor-ligand interactions may also contribute to the distribution of sTEM8 and its variant L56A. TEM8 is expressed in the respiratory epithelium of the bronchi, especially in the ciliated epithelial cells surrounding the luminal surface, the smooth muscle cells surrounding the vessels, and the epithelial cells lining the alveoli but is only weakly expressed in the endothelial cells lining the pulmonary vessels, as determined by immuno-histochemistry in mice using an anti-TEM8 polyclonal antibody that specifically recognizes the long-form of TEM8 localized at the cell surface [23]. Immunohistochemical studies have also shown the expression of CMG2 in the epithelial cells lining the skin, colon, and lung and in the vascular endothelium of these tissues in mice [24], [25]. Further studies have used real-time RT-PCR to show that both TEM8 and CMG2 are expressed in the lung, with TEM8 being more highly-expressed, but neither are expressed in the kidney [26]. Considering their homology, TEM8 and CMG2 may also be expressed as cell receptors in the lungs of rats. The concurrence between the target organ and the organ-specific expression may imply that organ-targeting may be receptor-ligand interaction dependent. This assumes that the physical ligand would always be present near its receptors. In the pharmacokinetic study, this receptor-ligand interaction may result in some non-linear characteristics, which may require further examination. The physiological functions of TEM8 and CMG2 remain unclear. However, the vWA domain of CMG2 has been reported to bind to basement-membrane matrix proteins, collagen type IV, and laminin [20], whereas TEM8 binds to collagen I and gelatin [27]. These matrix proteins are potential binding targets considering the reported co-localization of CMG2 and collagen type IV [24]. However, they cannot explain organ-specific pooling because they are distributed extensively throughout the body. sCMG2 appeared to be incapable of lung-targeting, given that the spleen was the second organ target and the lung did not show any enrichment compared to other organs (Fig. 5). Given the conclusive evidence that TEM8 and CMG2 are expressed as cell surface receptors in the lungs of mice [23], [24], the targeting of sTEM8 and L56A to the lung may account for their superiority in protecting the lung from anthrax toxin attack.

The pharmacokinetic parameters based on the drug concentration-time curve showed that all three proteins distributed and were eliminated at a fast rate. The fast distribution rate constant α for L56A is in accordance with its elevated affinity over sTEM8, which supports the involvement of receptor-ligand interactions as a mechanism contributing to their tissue distribution, given that they share the same targeting motif and have only a single difference at residue 56, which is distant from the GFE motif. In this way, this hypothetical ligand may interact with the ATRs in much the same way as PA does. Moreover, with a much higher affinity, the value of α for sCMG2 is less than that for L56A, implying that sCMG2 exhibits a different mechanism for distribution or employs a different receptor-ligand interaction that is much more difficult to assess or has weaker binding. With regard to elimination, sTEM8 and L56A presented longer half-lives than did sCMG2, which may be ascribed to the plasma protein binding that protects the constructs from protease–mediated catalysis, and also to their tissue distribution to lung, which may function as a reservoir, instead of the kidney, which may facilitate clearance from the body.

Overall, plasma protein binding and lung targeting seem to confer superior efficacy to sTEM8 and L56A over sCMG2 *in vivo*, which may explain why their performance approached that of sCMG2 after i.v. administration, despite their inferior protection *in vitro*.

The superiority of receptor-like decoys over antibody-based antitoxins lies in their ability to accurately mimic the natural receptor independent of natural or artificial changes in the toxin's amino acids, which would easily incapacitate the ability of site-specific antibodies to block toxicity [14]. This characteristic indicates that decoy design strategies should emphasize competition with the physical receptor, with equal consideration placed on elevating affinity and improving target organ access. It has been reported that PA, together with LF and EF, are all rapidly degraded, with early localization of radioactivity in the liver, spleen, and intestines and excretion through the kidneys [28]. However, for anthrax, present evidence indicates that the lung, rather than the liver, is the organ that both expresses the relevant toxin receptors and is the chief focus of infection and pathogenesis [26], [29]. It is possible that PA that distributes to target organs at a dose under the level of detection may still be sufficient for intoxication, which may emphasize the importance of co-localizing decoys with the corresponding native receptors to block the receptors and not just the toxin in circulation. sTEM8 and its higher-affinity variant L56A, which is designed by homology exchange to preserve the natural receptor's character, could effectively target the lung and confer protection to rats with a performance approaching or even exceeding that of sCMG2. This is extremely unexpected given their relatively lower affinities for PA. However, their plasma residence times were still too short for drug applications. Given that Fc-fusion strategies have been applied to sCMG2, which effectively prolongs its half time while preserving its decoy function, similar strategies may be explored to develop prospective decoys based on sTEM8 or L56A with elevated affinities, prolonged plasma residence times and improved organ targeting in anthrax antitoxin research.

## Materials and Methods

### Plasmid construction, protein expression and purification

DNA fragments of the vWA domains of TEM8 (aa 38–220, GenBank Accession Number AF421380; denoted as sTEM8) and CMG2 (aa 38–218, GenBank Accession Number AY233452; denoted as sCMG2) were amplified from cDNAs maintained in our laboratory and cloned into PHAT vectors (EMBL, Heidelberg) between NcoI and BamHI sites with a six-His tag at the 5′ end. Mutant L56A was obtained by inverse polymerase chain reaction (PCR) using mutation-inducing primers at the base of sTEM8 [17]. All clones were then validated by sequencing. In all constructs, the Cys-Ala mutation (Cys 177 in TEM8 and Cys 175 in CMG2) was introduced to reduce dimer formation during protein expression.

The constructs were expressed in BL21 (DE3) strains. After growth for 4 h in 1 l of LB medium, the cell cultures were induced with 0.4 mM IPTG for 16 h at 16°C. After ultrasonication, the supernatants were separated using a Ni-affinity column (GE Healthcare). sTEM8 and L56A were changed into Tris buffer (pH 8.0) with 50 mM sodium chloride by ultrafiltration, after which the target peaks were polished using a Source30Q column (GE Healthcare). sCMG2 was changed into Tris buffer (pH 9.0) containing 50 mM sodium chloride, and the peaks were directly collected after being passed through a Source30Q column. Finally, all proteins were concentrated and changed into Tris buffer (pH 8.0) with 150 mM sodium chloride. The purity of the recombinant proteins was analyzed by SDS-PAGE followed by Coomassie Brilliant Blue staining. The protein concentrations were then determined by the BCA Protein Assay (Thermo Scientific, Rockford).

### Cell protection assay and Schild plot analysis

Mouse macrophage J774A.1 cells were plated at 30,000 cells/well in 96-well plates and cultured for 24 h before treatment. The cells were then pre-cooled to 4°C to stop endocytosis. For the cell protection assay, a dilution series of the constructs, combined with PA and LF (final concentration of 100 ng/ml each), was added to the cells to a final volume of 100 µl/well. For the Schild plot analysis, a dilution series of PA combined with LF (final concentration of 100 ng/ml) was added to the cells along with different concentrations of the constructs. Next, the plates were incubated at 4°C for 2 h for complete competition and then transferred to 37°C. Cell viability was assayed 4 h after treatment by replacing the medium with 100 µl of fresh medium (MEM plus 2% FBS) containing 1 mg/ml MTT (Invitrogen, USA). After 1 h of incubation at 37°C, the medium was removed and the blue pigment produced by the viable cells was dissolved in 50 µl/well of 0.5% (w/v) SDS and 25 mM hydrochloric acid in 90% (v/v) isopropanol. The plates were then vortexed, and oxidized MTT was measured as the absorbance at 570 nm using a Model 550 microplate reader (Bio-Rad, USA). Cell viability was calculated as a percentage using the equation (OD_mesured_−OD_death control_)/(OD_live control_−OD_death control_), where “live control” wells contain LF alone and “death control” wells contain both 100 ng/ml PA and 100 ng/ml LF. IC_50_ or EC_50_ values were determined by nonlinear regression sigmoidal dose-response analysis with variable slopes (Prism, version 4.0; GraphPad, USA). Each assay was performed at least three times, with duplicates within each assay. The model used to fit the probable mathematical relationship between affinity and inhibition concentration is detailed in Supporting Information S1.

### Anthrax lethal toxin (LeTx) challenge in rats

*In vivo* experiments with animals were performed according to previously published methods [30]. All experiments were approved by the Animal Care and Use Committee of the Beijing Institute of Microbiology and Epidemiology (permit numbers: 20100701 and 20101101). Male Fisher 344 rats (Vital River, China) weighing 200–250 g were challenged with LeTx (50 µg PA mixed with 25 µg LF to a final volume of 500 µl in PBS per rat) via the tail vein. For rats that received receptors or mutants, decoys were also added to LeTx to a final volume of 500 µl, and the resultant solution was co-injected into the rats. The rats were then monitored for intoxication symptoms and death. Statistical analysis was conducted using the logrank test (Prism, version 4.0).

### Radio iodination and purification of ^125^I-sTEM8, ^125^I-L56A and ^125^I-sCMG2

Iodogen (1,3,4,6-tetrchloro-3α,6α-diphenylglycoluril) and Na^125^I solutions were obtained from Sigma Chemical Co. (St. Louis, MO, USA) and Perkin Elmer Life Sciences Inc. (Boston, MA, USA), respectively. sTEM8, L56A, and sCMG2 contain several tyrosine residues that enable them to be ^125^I-labeled [31]. sTEM8, L56A, and sCMG2 were radiolabeled with Na^125^I using the Iodo-Gen method as described in the manual. Briefly, 1 ml of each protein (2 mg/ml) was incubated with 50 µl of Na^125^I (5 mCi) in a reaction tube coated with 100 µg of iodogen at room temperature with gentle stirring for 10 min. The incubation was stopped by the addition of PBS (20 mM, pH 7.4). After incubation, to separate free ^125^I from the protein-bound ^125^I, the iodinated protein was purified on a Sephacyl™ S-200 high-resolution column (1 cm×40 cm) by eluting with PBS (20 mM, pH 7.4) at a flow rate of 1 ml/min. The effluents were collected at 1-min intervals. Through radioactivity determination, the fractions containing ^125^I-labeled protein were collected, and the remaining fractions were discarded. Under size exclusion chromatography conditions, ^125^I-sTEM8, ^125^I-L56A, and ^125^I-sCMG2 were all eluted between 9 to 12 min, whereas free ^125^I was eluted between 16 to 18 min after injection. The radiochemical purity of the ^125^I-labeled protein was confirmed to be greater than 95% as determined by HPLC. The proteins labeled with ^125^I had a specific activity of 198.19 kBq/µg for sTEM8, 141.23 kBq/µg for L56A and 56.67 kBq/µg for sCMG2.

### Validation of radioactivity determination in serum, urine, and tissues by TCA precipitation assay

Precipitation of the iodinated proteins in the plasma and tissues by ice-cold 10% TCA was used to remove free ^125^I or ^125^I associated with the fragmented peptides. Hence, TCA-precipitable radioactivity rather than total radioactivity was used to calculate the ^125^I-protein concentration in rat serum and tissue homogenate samples. A series of calibration standards were prepared by adding five concentrations of 0.045–11.528 µg/ml for ^125^I-L56A, 0.054–33.7 µg/ml for sTEM8, and 0.03–16.08 µg/ml for sCMG2 into the blank serum, tissue and urine samples that were examined. The relationship between the added concentrations and measured radioactivity of the standards was evaluated. The results showed good relationships (r^2^>0.99) and recovery (>80%) for the entire matrix.

### Pharmacokinetic studies

Rats (n = 6, 200±20 g) were i.v. injected with ^125^I-L56A, ^125^I-sTEM8, and ^125^I-sCMG2 at a dose of 67.5 g/kg via the tail vein (at volume 250 µl). Blood samples (approximately 200 µl each) were collected from the tail vein at 0, 5, 10, 20 min, and 0.5, 1, 2, and 4 h from the same rat and then centrifuged at 3000 g for 10 min. Serum samples at 5 min, 30 min, and 4 h after dosing were analyzed by size exclusion HPLC (TSK G3000 SWXL gel column, 300 Å, 10 µm, 7.8 mm×300 mm)-flow scintillation analyzer (Radiomatic Model 600TRSeries, PerkinElmer, USA) to investigate the metabolism of each protein *in vivo*.

^125^I-protein-associated radioactivity in the serum samples was determined after precipitation with TCA (10%, v/v). Briefly, each serum aliquot (50 µl) was added to 0.4 ml of ice-cold TCA (10%, v/v), vortex-mixed, and incubated on ice for 30 min. The mixture was then centrifuged at 16,000×g at 4°C for 10 min, and the supernatant, containing free ^125^I or ^125^I associated with fragmented peptide, was aspirated from each sample. The resultant TCA precipitate was then counted using a gamma counter (2470-005, WIZARDTM PerkinElmer, Finland) to determine the amount of ^125^I radioactivity that remained associated with precipitable protein. The result of ^125^I-labeled protein concentration in each time point was expressed as nanogram equiv. per milliliter (ng·equ·mL^−1^). A two-department model was applied, and the data were fitted using a two-phase exponential decay equation (GraphPad Prism software, San Diego California USA) with constraints of α>1, β>1, and values shared.

### Tissue distribution of ^125^I-L56A, ^125^I-sTEM8, and ^125^I-sCMG2

Three groups of rats (n = 6 per group) were i.v. injected at a single dose of 67.5 g/kg in the same manner as described above. The rats in the three groups were sacrificed by decapitation at 5 min, 0.5, and 4 h post-dosing, and blood and urine samples (300–400 µl) were immediately collected. Blood aliquots were immediately processed, and the resultant serum samples were analyzed as described. The tissues or organs including the heart, lung, liver, spleen, kidney, bladder, testis, jejunum, adipose, muscle and brain were excised, trimmed of extraneous fat, residual muscle and connective tissue, thoroughly rinsed of residual blood or contents with ice-cold 20 mM PBS (pH 7.4), and blotted dry. For radioactivity assays, small slices of tissues/organs were individually weighed, recorded and immediately homogenized twice at 8000 rpm for 30 s each time in 400 µl of ice-cold TCA (10%, v/v). The homogenates were then centrifuged at 3000 rpm for 10 min at 4°C, and the pellet was used for counting in the gamma counter.

## Supporting Information

Supporting Information S1 In vitro inhibition cell model.
(DOC)
