# Supplementary material for: Tumor Endothelium Marker-8 Based Decoys Exhibit Superiority over Capillary Morphogenesis Protein-2 Based Decoys as Anthrax Toxin Inhibitors
Source: PLoS One. 2011 Jun 2;6(6):e20646. doi: 10.1371/journal.pone.0020646 (PMC3107238; doi:10.1371/journal.pone.0020646)
Supplement: Supporting Information S1 — In vitro inhibition cell model. (DOC) [file pone.0020646.s001.doc]

**Supporting information**

**S1. *In vitro* inhibition cell model**

It has been reported that the vWA domain of the anthrax toxin receptors may inhibit cell intoxication [1, 2]. By competing with cell receptors for PA, the affinity of the receptor decoys correlates with inhibition efficacy. Here, we constructed a model to fit the probable mathematical relationship between affinity and inhibition concentration. This model was built based on two main assumptions. First, the interaction between PA and its receptors can be regarded as freely diffusing in liquid and reaching equilibrium before the toxin-receptor complex is endocytosed. Second, receptors in the process of interacting with PA, including binding, polymerization, and endocytosis, can be regarded as one type of putative receptor.

In our model, soluble receptors administered at concentration X (denoted as Rm, with dissociation constant Kdm) compete with the cell receptors (denoted as R, with dissociation constant Kd, and quantified as [Rc]) for PA at concentration Y. In accordance with our assumptions, there exist two reactions: PA+Rm$\xleftrightarrow{Kdm}$PARm and PA+R$\xleftrightarrow{Kd}$PAR, and at equilibrium, these equations are applicable:

(1) $X=[Rm]+[PARm]$

(2) $Y=[PARm]+[PAR]+[PA]$

(3) $\frac{[PA][Rm]}{[PARm]}=Kdm$

(4) $\frac{[PA][R]}{[PAR]}=Kd$

(5) $[Rc]=[PAR]+[R]$

Rearrangement of the equations gives: $Y=\frac{Kd[PAR]}{Kdm([Rc]-[PAR])+Kd[PAR]}X+\frac{Kd[PAR]}{[Rc]-[PAR]}+[PAR]$, where [PAR] is related to cell death. In an experiment with a constant cell death rate such as 50%, [PAR] is fixed accordingly, as well as [Rc]. When applied to a classic Schild plot assay using the receptors as antagonists, the apparent EC_50_ values of PA are linear as receptor concentration increases. There exists the equation 1/slope=$\frac{kdm}{kd}*\frac{[Rc]-[PAR]}{[PAR]}$+1, where $\frac{[Rc]-[PAR]}{[PAR]}$ is a constant for a certain type of cell and simply equals 1 when EC_50_ is applied in the classic Schild method.

An enzyme-linked immunosorbent assay (ELISA) has been constructed to evaluate toxin-receptor binding and binding inhibitors [3]. However, the native receptor at the cell surface featured in the cell protection assay, in contrast to plate-coating recombinant protein, is superior for modeling *in vivo* toxin-receptor binding and decoy inhibition. The Schild plot assay has also frequently been used to obtain the apparent Kd of PA-receptor interactions [4, 5]. Here, the introduction of relative IC_50_ with the addition of two references for the cell protection assay, and the deduced relation between KD and IC_50_, together with the discussion of the receptor-like antagonist based Schild plot assay, would reduce the errors induced by the discrimination of cell numbers and receptor-expressing states of different experimental repeats and provide a more precise quantification for evaluating receptor-like inhibitors.

**References**

1. Scobie HM, Thomas D, Marlett JM, Destito G, Wigelsworth DJ, et al. (2005) A soluble receptor decoy protects rats against anthrax lethal toxin challenge. J Infect Dis 192: 1047-1051.
2. Sharma S, Thomas D, Marlett J, Manchester M, Young JA (2009) Efficient neutralization of antibody-resistant forms of anthrax toxin by a soluble receptor decoy inhibitor. Antimicrob Agents Chemother 53: 1210-1212.
3. Dawson RM (2009) The CMG2 ELISA for evaluating inhibitors of the binding of anthrax toxin protective antigen to its receptor. J Pharmacol Toxicol Methods 59: 50-55.
4. Liu S, Leung HJ, Leppla SH (2007) Characterization of the interaction between anthrax toxin and its cellular receptors. Cell Microbiol 9: 977-987.
5. Chen KH, Liu S, Bankston LA, Liddington RC, Leppla SH (2007) Selection of anthrax toxin protective antigen variants that discriminate between the cellular receptors TEM8 and CMG2 and achieve targeting of tumor cells. J Biol Chem 282: 9834-9845.
